# Supplementary figures and images for: Identification of Runs of Homozygosity Islands and Functional Variants in Wenchang Chicken
Source: Animals (Basel). 2023 May 15;13(10):1645. doi: 10.3390/ani13101645 (PMC10215771; doi:10.3390/ani13101645)

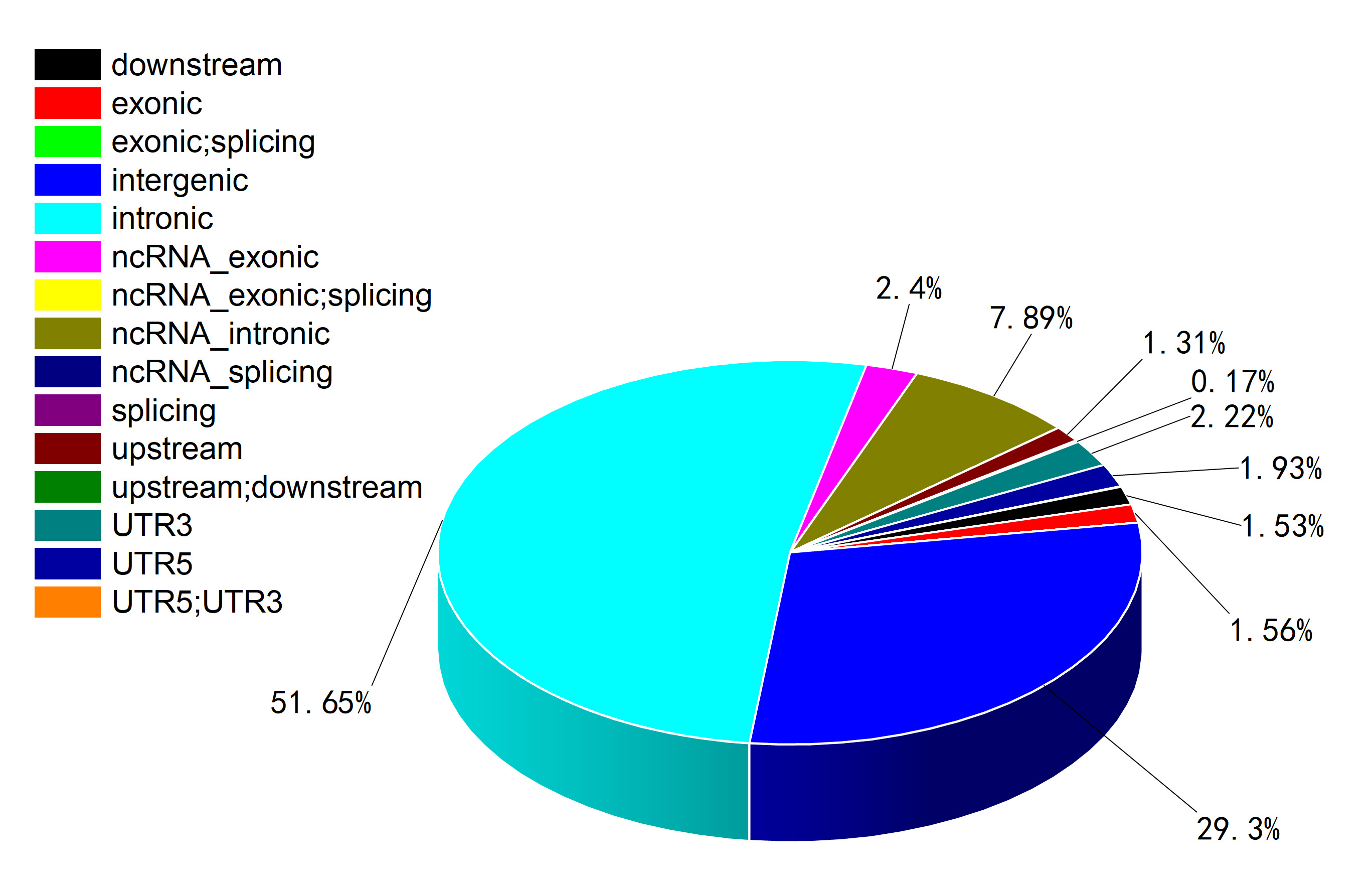

Supplement: Supplementary file 1 [file animals-13-01645-s001.zip › FigureS1.jpg]

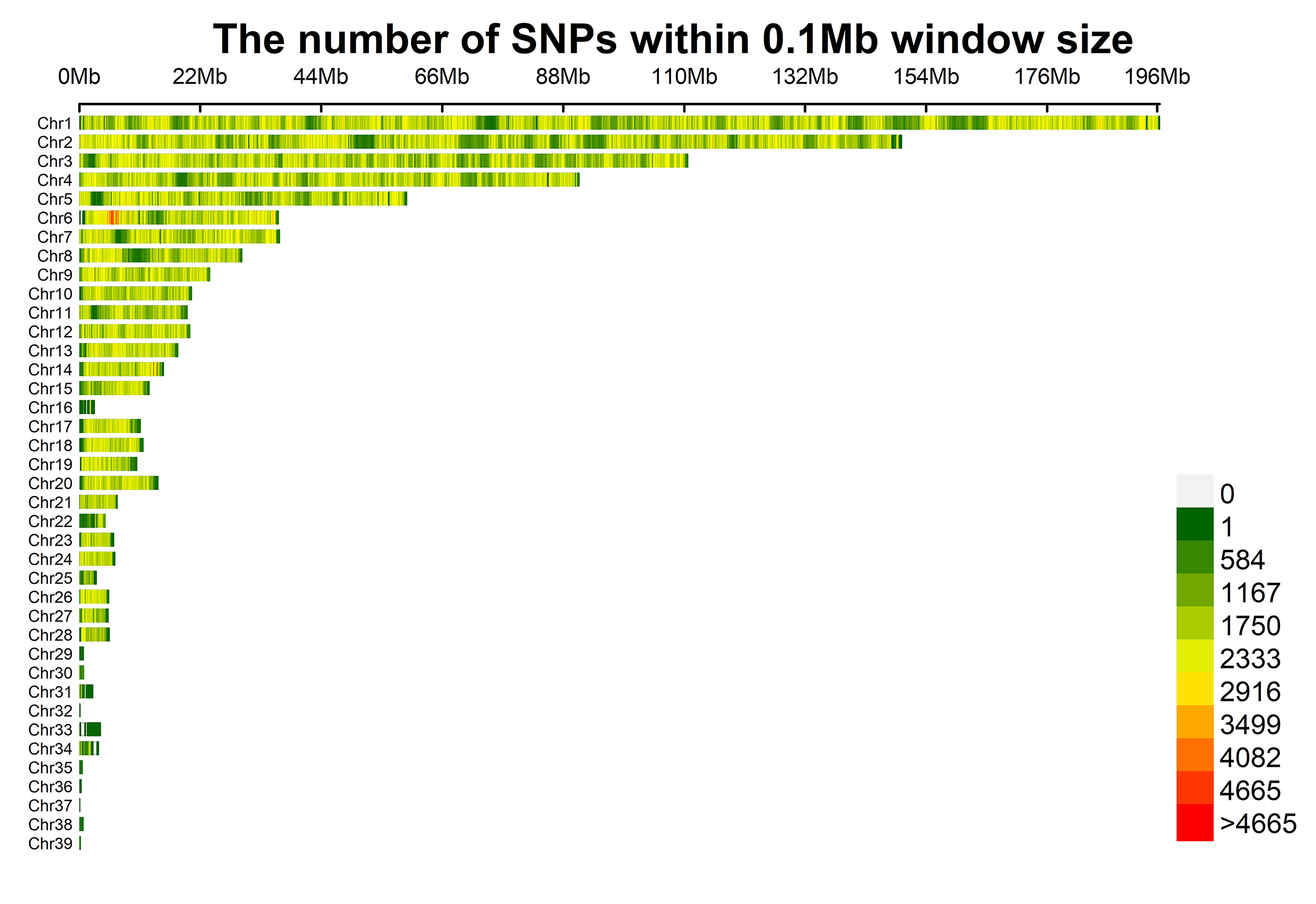

Supplement: Supplementary file 1 [file animals-13-01645-s001.zip › FigureS2.jpg]

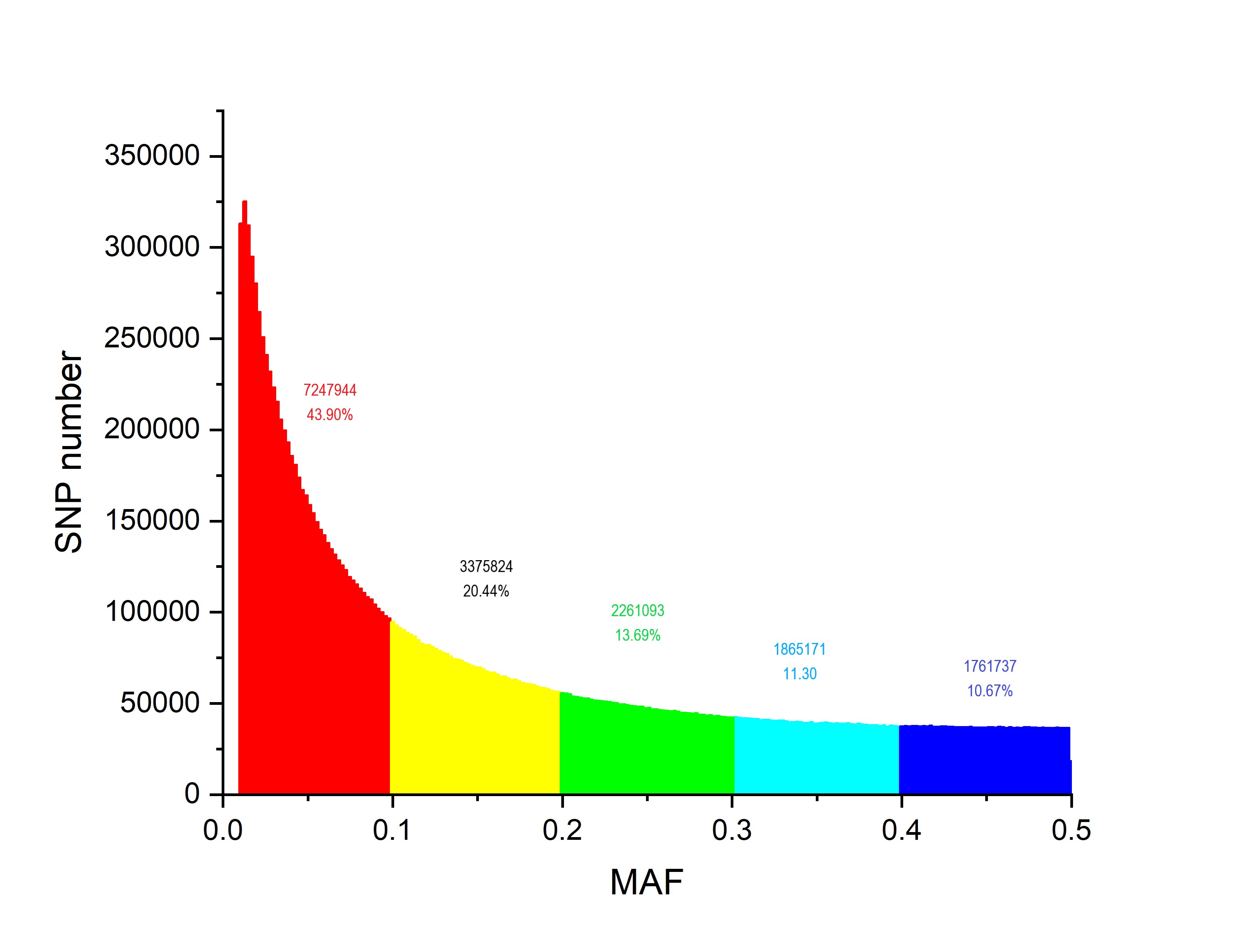

Supplement: Supplementary file 1 [file animals-13-01645-s001.zip › FigureS3.jpg]
